# Oxidative Stress Markers Differ in Two Placental Dysfunction Pathologies: Pregnancy-Induced Hypertension and Intrauterine Growth Restriction

**DOI:** 10.1155/2020/1323891

**Published:** 2020-06-30

**Authors:** Aleksandra Zygula, Przemyslaw Kosinski, Piotr Wroczynski, Magdalena Makarewicz-Wujec, Bronislawa Pietrzak, Mirosław Wielgos, Joanna Giebultowicz

**Affiliations:** ^1^1st Department of Obstetrics and Gynecology, Medical University of Warsaw, 1/3 Starynkiewicza Square, 02-015 Warsaw, Poland; ^2^Department of Bioanalysis and Drugs Analysis, Faculty of Pharmacy, Medical University of Warsaw, 1 Banacha Street, 02-097 Warsaw, Poland; ^3^Department of Clinical Pharmacy and Pharmaceutical Care, Faculty of Pharmacy, Medical University of Warsaw, 1 Banacha Street, 02-097 Warsaw, Poland

## Abstract

**Aim:**

Pregnancy-induced hypertension (PIH) and intrauterine growth restriction (IUGR) are both multisystemic disorders of pregnancy that cause perinatal morbidity and mortality. Recently, researchers focused on the role of oxidative stress (OS) as a pathophysiological mechanism in the development of these pathologies. The aim of this study was to compare OS in placental-related pathologies (PIH and IUGR) and uncomplicated pregnancies. We also investigated which salivary OS markers reflect systemic oxidative status and which only reflect the state of the oral cavity. *Material and Methods*. A total of 104 pregnant women (*n* = 104; 27 with PIH, 30 with IUGR, and 47 controls) were evaluated. Malondialdehyde (MDA), total antioxidant capacity (ORAC), aldehyde dehydrogenase (ALDH), and activity of glutathione peroxidase (GPx) and glutathione transferase (GST) in plasma/whole blood and/or saliva were analysed. Dietary nutrient intake was calculated using a Semiquantitative Food Frequency Questionnaire (SFFQ). Oral health was assessed to eliminate patients with bleeding, severe periodontitis, and other dental pathologies.

**Results:**

In the IUGR group, increased concentration of ORAC was observed both in saliva and plasma. Also, lower plasma levels of MDA in IUGR compared to the control group was detected. No sign of oxidative stress was confirmed in the PIH group. The examined groups did not differ regarding diet and markers of inflammation. ORAC in saliva was correlated with its level in plasma. No such correlations for MDA were observed. In the IUGR group, there were no differences in OS markers in plasma, but there was a lower ALDH level in the blood compared to the control group. It confirms OS occurrence in IUGR. In IUGR, a higher activity of salivary ALDH was probably due to worse oral health.

**Conclusion:**

Oxidative stress differs between IUGR and PIH groups: the presence of oxidative stress was confirmed only in the IUGR group. Salivary ORAC can be used to estimate ORAC in plasma. The activity of salivary ALDH reflects the state of the oral cavity.

## 1. Introduction

Placental dysfunction is a consequence of an inadequate remodelling of uterine and placental spiral arteries. Further sustained vasoconstriction of these arteries results in reduced transfer of oxygen to the foetus and subsequent foetal growth [1]. The resultant hypoxia or ischaemia, together with intermittent perfusion, is associated with reactive oxygen species (ROS) synthesis in the placenta [2, 3]. The villous trophoblasts and foetal-side blood vessels of terminal villi form a finely differentiated vascular network to serve the foetus with sufficient amounts of oxygen and substances for foetal growth [4]. The arterial circulation in the placenta is only regulated by local signals such as pressure and flow [5]. Abnormal maternal-foetal circulation due to poor placentation in early pregnancy makes the placenta hypoxic, and in response, a series of proinflammatory factors are released from the placenta that damage maternal endothelial cells; consequently, vascular resistance is increased, which as a result leads to maternal hypertension development and deteriorated placental angioarchitecture that may lead to abnormal foetal growth [6].

Oxidative stress suppresses nitric oxide (NO) bioavailability since it is rapidly degraded by the oxygen-derived free radical superoxide anion. The anion acts as a vasoconstrictor and is a major determinant of nitric oxide (NO) biosynthesis and bioavailability. Several studies have demonstrated that human hypertension is associated with a decrease of NO bioavailability and an excessive amount of ROS [7, 8]. Growing evidence shows that pregnancy-induced hypertension (PIH) and intrauterine growth restriction (IUGR) are caused by abnormal placental implantation, and oxidative stress may play a key role in these pregnancy disorders. The pathophysiology of IUGR is multifactorial and in most cases related to either maternal, foetal, placental, infectious, or genetic pathology [9]. In most cases, IUGR is thought to be the consequence of placental dysfunction [10]. Similar findings were observed in pregnancies complicated with PIH. Although the exact cause of PIH is still unknown, the basic pathologic event is a vascular endothelial dysfunction that leads to impaired placental implantation. By definition, PIH is a pregnancy-related disorder characterised by new-onset hypertension after 20 weeks of gestation. Growing evidence in recent years shows that hypertension-related complications of pregnancy are the major cause of maternal and perinatal morbidity and mortality [11].

The literature offers numerous reports on oxidative stress (OS) and placental dysfunction pathologies [12–20]. However, most of them are related to preeclampsia [6–8, 20], which is even more rare than pregnancy-induced hypertension (3-5% *vs.* 10%, respectively). It was shown that preeclampsia is associated with high levels of malondialdehyde (MDA), xanthine oxidase, and glutathione peroxidase and decreased levels of total antioxidant capacity and glutathione [6–9]. However, data on pathologies such as PIH [21–24] or IUGR [10–12] are rare.

The OS biomarkers can be found not only in blood but also in placenta [25], amniotic fluid [26], cord plasma [27, 28], and urine [17]. A modern diagnostic approach is to offer less invasive or even noninvasive tests for a diagnosis of different pathologies, and saliva seems to be a suitable material. Most of all, saliva has many advantages over blood, providing a cost-effective approach for the screening of large populations as it does not clot and can be more easily collected, transported, and stored. It is also safer in terms of potential infectious hazards [29]. There is a limited number of reports on the changes in the antioxidant profile, i.e., the concentrations of uric acid, MDA, and vitamins C and E; the activity of salivary peroxidase, salivary aldehyde dehydrogenase, and superoxide dismutase; and total antioxidant capacity in the saliva of pregnant women; however, to the best of our knowledge there are no studies on saliva from PIH and IUGR patients [30–32].

Our study describes antioxidant defence system and OS markers; however, dietary data was also used for the correct interpretation as oxidative stress markers can be severely influenced by diet. We aimed to determine whether the oxidative status of abnormal placental function-related pathologies (PIH, IUGR) and healthy controls differ noticeably, and whether any differences were visible between PIH and IUGR. Additionally, in this study, we investigated which among the salivary OS markers reflect the systemic OS and which only reflect the state in the oral cavity.

Placental tissue can be an important source of lipid peroxidation products in pregnancy, especially in the first trimester. This is because ROS formed in the placenta have easy access to membrane phospholipids, which are highly abundant at the site [33]. Thus, in our study, we focused on enzymes important in relieving the OS effects on lipids like glutathione transferase (GST), aldehyde dehydrogenase (ALDH), and glutathione peroxidase (GPx). The phase I metabolism of aldehydes generated during lipid peroxidation is carried by ALDH (aldehydes are oxidised to acids). In the phase II metabolism, GST catalyses the conjugation of aldehydes with GSH [34]. Finally, GPx reduces the lipid hydroperoxides. We have also determined the levels of the lipid peroxidation marker (MDA) and the total antioxidant capacity (ORAC).

## 2. Materials and Methods

The OS parameters and the antioxidant defence systems in two diagnostic materials were determined: standard (blood: plasma and whole blood) and alternative (saliva). The following were determined in blood: (1) antioxidant enzyme activity (glutathione peroxidase (GPx) and glutathione S-transferase (GST) in plasma; aldehyde dehydrogenase (ALDH) in whole blood), (2) total antioxidant capacity (ORAC) in plasma, and (3) malondialdehyde (MDA) levels in plasma. The following were determined in saliva: (1) activity of salivary ALDH, which protects the oral cavity from aldehydes (from food, generated in OS), (2) ORAC, and (3) MDA concentrations. Since diet influences OS, dietary nutrient intake was calculated using a Semiquantitative Food Frequency Questionnaire (SFFQ). Oral health was assessed to eliminate patients with bleeding, severe periodontitis, and other pathologies. The inflammation markers as C-reactive protein (CRP) and white blood cells (WBC) were also assessed.

### 2.1. Study Group

Fifty-seven pregnant women with pregnancy pathologies (PIH = 27, IUGR = 30) and 47 women with uncomplicated pregnancy (between 24 and 41 weeks of gestation) were enrolled. The study was conducted between January 2011 and January 2013. Gestational age was established based on the date of the last menstrual period and confirmed by first-trimester ultrasound scan. The inclusion criteria were as follows: singleton pregnancy and gestation age between 24 to 41 weeks. The exclusion criteria were as follows: multiple pregnancy, foetal abnormalities, chronic hypertension, autoimmune diseases, gingivitis, and smoking.

Controls were selected randomly and matched with the examined patients for maternal and gestational age, gravidity, parity, and BMI. The inclusion criteria for the control group were as follows: blood pressure (BP) equal to or lower than 140/90 mmHg after 20 weeks; age: 19-40 years; and no history of prepregnancy hypertension, diabetes, and concomitant systematic diseases. The diagnosis of IUGR was made when estimated foetal weight was below the 10th percentile and associated with abnormal foetal Doppler parameters. Women with proteinuria greater than 300 mg/day and history of prepregnancy diabetes, renal disease, autoimmune disorders, and smokers were excluded from both the control group and the study group. Moreover, patients with hypertensive disorders were excluded from the IUGR group. All PIH women were treated with methyldopa tablets as a first line therapy with a dose depending on blood pressure values (dosage: 0.5 g to 2 g). In all cases of poor blood pressure control, labetalol or metoprolol was introduced as a second line therapy. During hospitalisation, blood pressure was measured every 4 hours with an automatic blood pressure machine. All patients included in the study had regular medical check-ups at the First Department of Obstetrics and Gynecology, Medical University of Warsaw. Local Ethics Committee approved the protocol of this study (No. KB78/2011). All patients gave their written informed consent.

### 2.2. Sample Collection

Blood and saliva samples were collected in the morning after an overnight fast (at least 6 hours). All blood samples were collected into test tubes with ethylenediaminetetraacetic acid (EDTA) as an anticoagulant. Whole saliva samples and blood samples were obtained simultaneously between 8 and 9 am. Patients were asked to rinse the mouth with warm water, sit, relax, and rest for 5 minutes before sample collection. Saliva was allowed to accumulate in the floor of the mouth, and the patient spitted it into test tubes with 50 mM phosphate buffer, pH 7.5, with the addition of 0.5 mM EDTA and 1 mM GSH (ALDH activity) or without (ORAC, MDA). The samples were immediately centrifuged (saliva: 10,000 g, 10 min; blood: 3,000 g, 10 min). Plasma was separated, aliquoted, and stored at -80°C until analysed. Salivary supernatant was analysed up to 2 h after the collection for ALDH. Other saliva aliquots were stored at -80°C till further analysis. The storage of materials did not exceed 30 days.

### 2.3. Biochemical Analysis

Results are the average of three independent measurements.

#### 2.3.1. Enzyme Activity

Total glutathione peroxidase activity (GPx) was measured in plasma spectrophotometrically, using the method developed by Paglia and Valentine and modified by Wendel, at a wavelength of 340 nm [35]. The final reaction mixture contained glutathione (GSH, 1.0 mM, Sigma-Aldrich), a reduced form of nicotinamide adenine dinucleotide phosphate (NADPH, 65 *μ*M, Sigma-Aldrich), and sodium azide (0.17 mM, Sigma-Aldrich). The reaction was carried out at 25°C in 50 mM sodium phosphate buffer with 0.40 mM EDTA (Sigma-Aldrich), pH = 7.0. Cumene hydroperoxide (1.05 mM, Sigma-Aldrich) was used as a substrate.

Glutathione S-transferase activity (GST) was measured spectrophotometrically at a wavelength of 340 nm, using the method described by Habig et al. [36]. The final reaction mixture contained 2 mM GSH (Sigma-Aldrich) and 1 mM 1-chloro-2,4-dinitrobenzene (CDNB, Sigma-Aldrich) as a substrate. The reaction was carried out at 25°C in 50 mM sodium phosphate buffer with 0.50 mM EDTA (Sigma-Aldrich), pH = 7.5.

The GPx and GST activities were measured with a Synergy Mx microplate reader (BioTek). Salivary aldehyde dehydrogenase and blood aldehyde dehydrogenase (ALDH) activity were measured using the fluorometric method in the presence of 1 mM GSH or 0.5 mM DTT, respectively [37, 38]. Five *μ*M 6-methoxy-2-naphthaldehyde (Sigma-Aldrich) was used as a fluorogenic substrate, 100 *μ*M nicotinamide adenine dinucleotide (NAD^+^, Sigma-Aldrich) was used as a cofactor, and 6-methoxy-2-naphthoic acid (1.5 *μ*M) (Sigma-Aldrich) was used as a standard. The assay was also performed without the addition of NAD^+^ to eliminate nonspecific substrate oxidation. The excitation and emission wavelengths were set to 315 nm and 360 nm, respectively. The reaction rate was followed using an F-7000 Fluorescence Spectrophotometer (Hitachi). Blood ALDH activity was expressed as U/g of hemoglobin. Hemoglobin concentration was measured photometrically using Drabkin's reagent (Humana) and a Synergy Mx microplate reader (BioTek).

#### 2.3.2. ORAC Assay

The ORAC-fluorescein (ORAC) fluorometric assay was performed based on the procedure of Ou et al. [39]. Briefly, 70 *μ*l of PBS (phosphate-buffered saline, pH = 7.4, Sigma-Aldrich), 30 *μ*l of a diluted sample of PBS, and 100 *μ*l of a 112 nM sodium fluorescein (Sigma-Aldrich) solution were mixed in a well and thermostated for 15 min at 37°C. Then, 100 *μ*l of a 48 mM AAPH (2,2′-azobis(2-amidinopropane) dihydrochloride, Sigma-Aldrich) solution was added and fluorescence was measured every 60 s for 90 min with the excitation and emission wavelengths of 485 nm and 520 nm, respectively, on the F-7000 Fluorescence Spectrophotometer (Hitachi). The ORAC values were expressed in Trolox equivalents. The ORAC assay is useful for investigating the effectiveness of chain-breaking antioxidants. The contribution of plasma antioxidants to the total value (in decreasing order) is as follows: proteins (sulfhydryl groups), uric acid, vitamin C, vitamin E, bilirubin, and carotenoids [40].

#### 2.3.3. MDA Concentration

The assay was based on the reaction of MDA with thiobarbituric acid (23 mM, Sigma-Aldrich) in the presence of 13 mM sodium dodecyl sulfate (SDS, Sigma-Aldrich), 3 mM EDTA (Sigma-Aldrich), and acetate buffer (pH = 3.5, Sigma-Aldrich) [41]. Reaction was carried at 95°C for 60 min, then cooled and centrifuged (5,000 g for 10 min). The supernatant was shaken with the same volume of butanol and centrifuged (5,000 g for 10 min). The organic layer was immediately analysed. Separation was performed on a C18 column (Kromasil C18, 250 × 4.6 mm, 5 *μ*m, Sigma-Aldrich) with a mixture of methanol (POCH) and Mill-Q water (1 : 1) as the mobile phase. The flow was set at 1 ml/min. The excitation wavelength was set at 515 nm and the emission wavelength at 535 nm. Injections of 50 *μ*l were performed. MDA was assayed on a high-performance liquid chromatograph (Shimadzu) equipped with two pumps (LC-10 AT VP and LC-10 AD VP), a column oven (CTO-10 A VP), a fluorescence detector (RF-10 A XL), a degasser (DGU-14A), and a system controller (SCL-10A VP).

### 2.4. Diet

Dietary nutrient intake was calculated using SFFQ, consisting of a list of foods with standard serving sizes commonly consumed by the Polish adult population. The participants were asked to report how often they consumed each of the food items listed (number of times per day, per week, per month) during the previous month. The reported frequency for each item was then converted to a daily nutrient intake. Portion sizes of the consumed foods were converted to grams using household measures. The nutritional value of the diet was calculated using the “Dieta 5” software developed by the Food and Nutrition Institute.

### 2.5. Oral Health

Each patient received a complete oral and periodontal examination. The controlled oral health indicators are shown in Table 1. In this study, the regular plaque index determination was modified. The teeth were examined for the presence of any plaque or calculus, and clean teeth were marked as 0, while teeth with any plaque as 1. The final value was the sum of all teeth with plaque or calculus. The patients were also assessed for periodontal lesions or prosthetic restorations. All patients had healthy and lesion-free oral mucosa. Four patients used nonremovable restorations (prosthetic bridge or crown), one patient used removable prosthesis, and one had an orthodontic retainer.

### 2.6. Statistical Analysis

Statistical evaluation of the results was performed with Statistica version 13.1 for Windows. Normal distribution of the results was evaluated by the Shapiro-Wilk test. In case of normal distribution, ANOVA was used, otherwise the Mann-Whitney *U* and Kruskal-Wallis one-way tests were applied. Differences between nominal variables were tested using the Chi-square test. Correlations were presented as the Spearman correlation coefficients. To identify the risk factors of IUGR and PIH, the univariate and backward elimination logistic regression analyses were performed and odds ratios (OR) and 95% confidence intervals (CI) were estimated. The discriminating power of different parameters was analysed by means of Receiver Operating Characteristic (ROC) curves. The *p* value below 0.05 was considered as significant.

## 3. Results

### 3.1. Characteristics of the Study Population

Clinical characteristics of the study group are presented in Table 2. Mean maternal age in the study group was 29 years (min 19, max 38 years). No statistically significant differences between the study groups were found regarding age, parity, gestational age, body mass index, and Apgar score. Besides lower foetal weight, no differences were found between the PIH and control groups. The prevalence of cavities, missing teeth, and increased gingival index was higher in the IUGR patients compared to the other groups.

### 3.2. Diet

Basic dietary parameters for the entire study population are presented in Table 3. No significant differences in the diet between the PIH, IUGR, and control groups were observed. The similar intake of energy, flavonoids, fibre, vitamin C, heme iron, and total iron at +2 oxidation state was noted.

### 3.3. Biochemical Analysis

Median activity of GPx and GST, value of ORAC, and concentration of MDA in plasma and/or saliva as well as activity of ALDH in blood and saliva are presented in Table 4. No significant differences were observed in OS markers between PIH and controls (Table 4). The results were confirmed using regression analysis. Even GPx (OR = 1.070, CI: 1.003-1.143, *p* = 0.0409) was identified from univariate analysis as a significant risk factor of PIH; ROC curve analysis revealed no class separation capacity (AUC = 0.51 for the validation data set).

In the IUGR group, we observed lower blood but higher salivary ALDH activity than in the PIH and control groups. The IUGR group also had an elevated concentration of ORAC both in saliva and plasma and had lower plasma levels of MDA than the control group (Table 4). Similar results were obtained using regression analysis. Significant risk factors of IUGR identified from univariate analysis were ALDH1 (OR = 0.820, CI: 0.698-0.964, *p* = 0.0097), salivary ORAC (OR = 1.494, CI: 1.140-1.958, *p* = 0.0036), and vitamin E (OR = 0.781; CI: 0.662-0.923, *p* = 0.0007) intake. However, in multivariate analysis, salivary ORAC (OR = 1.485, CI: 1.081-2.041, *p* = 0.015) and interaction between salivary ORAC and ALDH1 (OR = 0.829, CI: 0.700-0.983, *p* = 0.031) were the only independent factors in determining IUGR. The discriminative power expressed as ROC area was 0.72 (for the validation data set).

**Figure 1:**
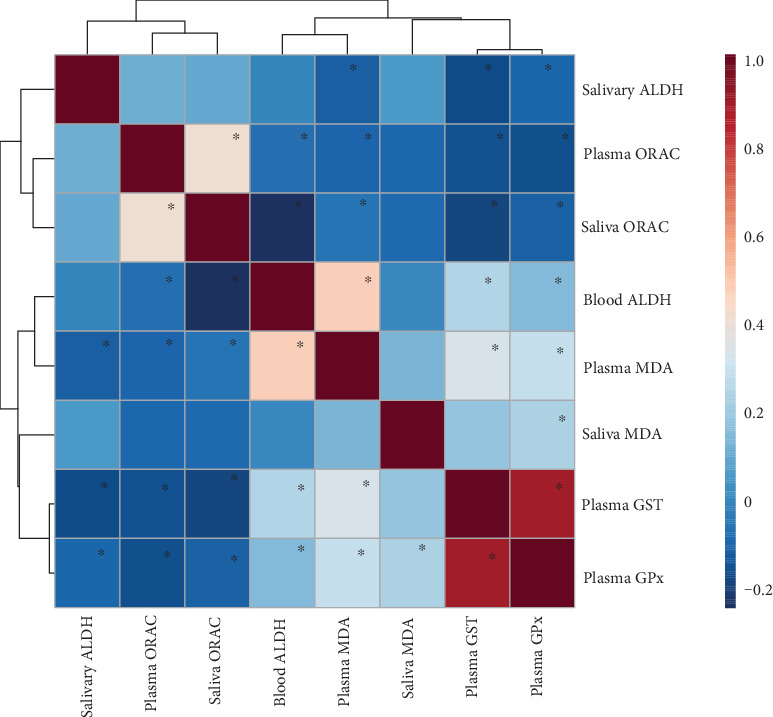
Correlation pattern (Spearman's correlation) of the activity of the enzymes, concentration of malondialdehyde, and ORAC value in blood and saliva. ^∗^Significant correlations (*p* < 0.05). Different colours indicate different Spearman's correlation coefficients (strength and direction of correlation), i.e., red colour—positive correlation and blue colour—negative correlation; the darker the colour, the higher the strength of the relationship. Abbreviations: ALDH—aldehyde dehydrogenase; ORAC—oxygen radical absorbance; MDA—malondialdehyde; GST—glutathione S-transferase; GPx—glutathione peroxidase.

Correlations between the biochemical parameters are presented in Figure 1. There was a correlation of ORAC in plasma and saliva. The relation was observed for all examined groups (*r* = 0.53, *p* < 0.0001), for the control group (*r* = 0.43, *p* = 0.0067), for the PIH group (*r* = 0.56, *p* = 0.0061), and for the IUGR group (*r* = 0.46, *p* = 0.0477). No correlation was observed for MDA in plasma and saliva, neither in all examined groups nor in all subgroups.

## 4. Discussion

It is hypothesised that oxidative stress, defined as oxidant-antioxidant imbalance, has a pivotal role in the development of placental-related disorders, such as hypertensive disorders of pregnancy and IUGR [42]. In our study, the PIH group had comparable activity of antioxidant enzymes in plasma, whole blood, and saliva; comparable level of lipid peroxidation markers (MDA); and similar ORAC to the control group. It should be highlighted that the PIH group consisted only of pregnancy-induced hypertension developed after 20 weeks of pregnancy (no preeclampsia and no proteinuria above 300 mg/24 h were noted). The occurrence of OS in preeclampsia was thoroughly studied and confirmed before. The elevated level of OS markers such as MDA and 8-hydroxy-2′-deoxyguanosine (8-OHdG) and the decreased level of total antioxidant capacity were described by several researchers [11, 15, 20, 37]. Data on the pregnancy-induced hypertension is rare [21–24], but it also suggests the occurrence of OS. It should be highlighted that the comparison of OS between two groups of patients is challenging. It is due to many factors that influence oxidative status, with comorbidity and diet being the most important [43, 44]. Thus, in our study, we decided to analyse not only antioxidant parameters but also diet and markers of inflammation (white blood cells (WBC), C-reactive protein (CRP)). It is already well known that diet has a great impact on OS markers and antioxidant capacity [44, 45]. High consumption of macronutrients can trigger oxidative stress and subsequently contribute to inflammation *via* NF*κ*B. Dietary carbohydrates of high glycaemic index may particularly contribute to long-term consequences of nutrition-related inflammation. It is well documented that long-chain saturated fatty acids may promote proinflammatory endothelial cell phenotypes. On the other hand, fibres, several vitamins, flavonoids, and short-chain fatty acids were shown to decrease ROS levels. Flavonoids and vitamins react with and inactivate ROS, whereas fibres influence gut microbiota, which have influence on immune function, e.g., through the production of short-chain fatty acids. Some particular compounds may inhibit oxidative stress and inflammation [43, 45]. High doses of iron at +2 oxidative state can also induce lipid peroxidation by the generation of hydroxyl radicals in a Fenton reaction [46].

Our study group with PIH was comparable with the control group not only regarding gestational age, CRP, WBC, and oral health but also with the diet. As mentioned previously, several research papers confirmed OS in patients with PIH; however, this was not the case in our study. There are few possible reasons why in our research no OS was noted within the PIH group. The first possible explanation is a different diet between the study group and the control group in other studies. In the studies concerning OS in pathologies such as PIH, which were published so far, the diet was not analysed. Another explanation is the differences in exposure to environmental stressors (i.e., UV and ionizing radiations, pollutants, and heavy metals), lifestyle factors such as physical activity, and dietary habits (some ingredients can better neutralize ROS generated during OS caused by the disease than others) among studies [47, 48]. Finally, despite that no sign of oxidative stress in PIH in our study was observed, we cannot exclude the potential role of OS in the pathogenesis of PIH. Our OS profile analysis included only antioxidant enzymes important for relieving the OS effects on lipids (GPx, GST, and ALDH) and lipid peroxidation markers (MDA) and the total antioxidant capacity (ORAC). However, there are many other OS markers such as the markers of protein damage (e.g., oxidized protein and protein carbonyl content) and the markers of DNA damage (e.g., 8-hydroxydeoxyguanosine) as well as various antioxidant enzymes with the most popular being catalase and superoxide dismutase, whose activity can be affected in PIH. Thus, further studies are needed including not only biochemical analysis and careful clinical examination but also surveys using questionnaires that gather data on dietary habits, exposure to environmental stressors, and physical activity.

The sign of oxidative stress was observed in IUGR. Although no differences in the activity of antioxidant enzymes were found in plasma, we observed differences in whole blood. The data on antioxidant status in plasma and whole blood will provide different information. By analysing plasma, we can determine the actual antioxidant status of patients. Red blood cells (RBC) that live for 100-120 days have a restricted ability to respond to oxidant stress; due to lack of nuclei, it cannot synthesize new proteins or replace irreversibly damaged cellular components [49]. Thus, analysing RBC gives us some information about the past. We observed 2-times lower activity of ALDH in the IUGR group than in the control group, suggesting severe OS in the past. When OS is mild to moderate, the reversible oxidation of thiols (Cys-SH) to sulfenic acid (Cys-SOH) or their nonreversible oxidation to sulfinic (Cys-SO_2_H) or sulfonic (Cys-SO_3_H) acids in Keap1 occurs. As a result, Nrf2 is released from the Nrf2-Keap1 complex. Keap1 binds IKK*β* and suppresses the nuclear factor-kappa B (NF*κ*B) activation and inflammatory response. The released Nrf2 upregulates Antioxidant Responsive Element (ARE) genes, like ALDH. Enhanced expression of antioxidant enzymes coded by ARE-responsive genes reduces OS and restores the reductive environment of cells. Keap1 binds to Nrf2 and inhibits its interaction with ARE. But when OS is severe, Nrf2 can promote ROS generation. The oxidation of Keap1 is then not reversible, leaving Keap1 unable to revert to a protein-binding conformation. The inhibition of IKK*β* by Keap1 is abrogated, and NF*κ*B becomes active. NF*κ*B has been known to inhibit Nrf2 as well, so in heavy OS, the ARE genes are not upregulated anymore. The proteins produced as a result of NF*κ*B induction include the following proinflammatory cytokines: tumour necrosis factor-*α* (TNF-*α*) and IL-6 (which induces CRP production) [50].

The cause of maternal oxidative stress in IUGR is still not clear. However, inadequate perfusion leads to placental hypoxia at the intervillous space and may contribute to both maternal and foetal oxidative stress. Considerable remodelling of the placenta is observed at the end of the first trimester or at the start of the second trimester, which usually is the starting point for IUGR. Minor deficiencies in arterial conversion may lead to low-grade fluctuations in villous oxygenation that cause homeostatic responses in the form of mild endoplasmic reticulum stress and protein synthesis inhibition that leads to the small size of the placenta and reduced functional capacity [1, 51]. Since in IUGR (without coexisting hypertension) no sign of inflammation was noted, i.e., the levels of TNF-*α*, IL-6, and IL-8 were comparable to normal controls, chronic oxidative stress in the pathology is not very probable [52]. Major deficiencies in arterial conversion can cause more severe, and more frequent, placental ischaemia-reperfusion and can cause severe oxidative stress and inflammation resulting in IUGR presenting with hypertension. The oxidative stress and inflammation can be induced on top of the preexisting endoplasmic reticulum stress depending on, e.g., diet or enzyme polymorphism, and can lead to IUGR and coexisting hypertension as well [1, 51]. In our study, only patients with diagnosed IUGR without hypertension were included and no oxidative stress in plasma was noted. Similar results were obtained by Celik et al., who compared the oxidative stress index and the total antioxidant and total oxidant status in uncomplicated pregnancy and IUGR [53]. Others observed severe OS in IUGR patients. Increased protein carbonyls [54] and a higher level of plasma GPx [18] as well as reduced antioxidant capacity [54] and an elevated level of MDA in plasma patients with IUGR [17, 55] were observed. Similar to our research, the alteration of erythrocyte antioxidant enzymes in IUGR was noted. Saker et al. observed a reduced level of superoxide dismutase and catalase [54] (in our study, a decrease of activity of the other erythrocyte enzyme, ALDH, was noted). The increase of MDA and lipid peroxidation levels in the maternal erythrocytes was also observed, which can be a marker of severe OS [56, 57].

Differences between our results in IUGR and other results presented in the literature can be similar to those in the case of PIH. These differences are listed as follows: (a) In other publications, the diet was not analysed, and if the nutrition regime is not appropriate, it can influence oxidative stress in IUGR. (b) Several research studies were conducted after the delivery which can influence the oxidative status. (c) Some differences in exposure on environmental stressors (i.e., UV and ionizing radiations, pollutants, and heavy metals) and lifestyle factors such as physical activity and dietary habits (some ingredients can better neutralize ROS generated during OS caused by the disease than others) that influence OS among studies can be observed [47, 48]. (d) Patients with IUGR can be affected at different rates by environmental stressors; Pathak et al. suggested that higher levels of hexachlorocyclohexane (HCH) isomers (organochlorine pesticide) may be associated with IUGR and increased oxidative stress [58]. Unfortunately, we did not collect data on environmental stressors or physical activity. However, here we report the estimated values for Warsaw/Poland for future comparison. According to our survey, 35-50% of treated patients lived in the Warsaw agglomeration (Table 2). None of them drank alcohol or smoked cigarettes during pregnancy. According to recently published data, pregnant women from Warsaw perceived relaxation as a safer behaviour rather than regular exercise and maintaining an active lifestyle. Thus, physical activity was not satisfactory [59]. Regarding occupational risk, a high percentage of women in Poland take sick leave as early as <12 weeks of pregnancy because of conditions related to pregnancy. The number of such patients depends on the type of job they have and varies from 34% for the office workers to 68% for waitresses [60]. The exposure to organochlorine pesticides measured as the level in human milk was higher in Poland than in other European countries. The corresponding concentrations of DDE (dichlorodiphenyldichloroethylene), DDD (dichlorodiphenyldichloroethane), DDT (dichlorodiphenyltrichloroethane), and HCH were 241–12,803 *μ*g/l, 3–1,883 *μ*g/l, 35–3,055 *μ*g/l, and 6–203 *μ*g/l of milk, respectively [61]. The air over Poland is one of the most polluted in Europe. In the Warsaw population, the weighted exposure of NOx is 3.54 *μ*g/m^3^, PM (2.5)—16.84 *μ*g/m^3^, Pb—15 ng/m^3^, benzo(a)pyrene—1.95 ng/m^3^, and other metals (As, Cd, Ni)—6.1 ng/m^3^. The degree of pollution strongly depends on which region in Warsaw (with the highest values at the left side of the Vistula River) and what part of the year—with the highest pollution in the winter time [62].

In the IUGR group, a significantly higher activity of salivary ALDH was observed. Human salivary ALDH is the body's first line of defence in the oral cavity. It neutralizes aldehydes generated endogenously, e.g., as a result of oxidative stress, contained in food either as natural ingredients or as contaminants [63]. Higher salivary ALDH activity may be explained by the presence of the inflammation of the oral cavity in the IUGR group. Among the patients with IUGR, we observed a significantly higher gingival index, a number of teeth with cavities, and missing teeth. Several researchers noted that saliva from patients with periodontitis and/or dental cavities exhibited increased levels of oxidative stress as well as inflammatory markers [64, 65]. The ALDH expression is regulated by Nrf2. The stress can be classified as mild to moderate since only the induction of antioxidant enzymes was observed, without the changes in the lipid peroxidation marker MDA. So far, no influence of dental cavities on IUGR incidence was detected [66]. On the contrary, there is strong evidence that periodontal diseases can lead to IUGR since transient bacteraemia may facilitate dissemination of oral bacteria and/or proinflammatory mediators to the uterus, with subsequent infiltration of the amniotic fluid and the umbilical cord and invasion of the placenta. Some authors suggest that advanced periodontal diseases may be related to more frequent adverse pregnancy outcome including preterm birth [67, 68]. However, in our study group (where no signs of advanced periodontal disease were present), preterm delivery was not observed.

Interestingly, similar to Mert et al., we observed a higher ORAC level [19] in plasma of IUGR. Higher ORAC in IUGR was not related with differences in diet between groups. The intake of flavonoids and vitamins in the IUGR group in our study was comparable to that of the control group. The higher ORAC in IUGR can be the result of various metabolomic changes in maternal blood as a result of the pathology [69]. ORAC is one of the methods to measure total antioxidant status *in vitro*. ORAC level was shown to strongly correlate with uric acid concentration [70]. The compound is easily oxidized; thus, it serves as an antioxidant *in vitro* in the hydrophilic environment [71]. However, *in vivo*, it can reveal prooxidant action as well. When the uric acid reacts with oxidants, other radicals may be produced which might propagate a radical chain reaction and lead to oxidative damage to cells. Moreover, uric acid may be involved in intracellular oxidant production *via* the ubiquitous NADPH oxidase-dependent pathway resulting in redox-dependent intracellular signalling and, in some conditions, oxidative stress [72]. Thus, not all antioxidants *in vitro* serve as antioxidants *in vivo*, and the results should be interpreted with caution. In foetuses with IUGR, there is a strong evidence of a higher level of uric acid level in cord vein blood [73] and alteration in the compound in urine of neonates [74]. Uric acid together with pseudouridine and allantoin are metabolites involved in nucleoside metabolism [75]. Its increase may be related to the hypoxia or ischaemia, together with intermittent perfusion in IUGR. As an example, it was proven that uric acid changes in this marasmus model but it was not observed to change in kwashiorkor children [75]. Thus, further study should be performed to clarify the obtained results. Currently, we suppose that higher ORAC was not only related to metabolic changes. It reflected higher antioxidant content (not related to uric acid) in plasma in IUGR as well, especially since a lower MDA level in the group was observed. ORAC value is strongly relayed on protein content in the sample; thus, a high ORAC value can be a result of higher antioxidant enzyme activity and/or higher protein content [76]. A higher enzyme activity (other than the studied ones) and/or protein content can be a response to OS and inflammation. As an example, a higher ORAC was noted in children with dental cavities [77]. Thus, a higher ORAC in IUGR can be a compensatory mechanism to oxidative damage and serve as a marker of OS. Both the low level of ALDH in RBC (result of experienced OS) and the high level of ORAC (compensatory effect to experienced OS) suggest the role of OS in the pathogenesis of IUGR. Therefore, prospective studies designed to monitor the OS level from the first trimester of pregnancy to postpartum in patients with IUGR are required.

Saliva is an alternative material to blood. In our study, a significant correlation between ORAC in saliva and plasma was observed. Similar results were previously obtained for patients with gestational diabetes [78]. Saliva was also considered valuable for estimating the variation of total antioxidants in plasma of triathletes during the training season [79]. However, regarding lipid peroxidation markers (MDA), we observed no correlation in saliva and plasma. Similar results were observed previously among gestational diabetic patients [78] and it was concluded that the concentration of MDA in saliva is strongly dependent on the oxidative balance of saliva and the state of the oral cavity. However, in some pathologies related to strong oxidative stress, such correlation was observed. For example, in patients with recurrent aphthous ulcerations, both salivary and serum malondialdehyde (MDA) were elevated and strongly correlated (*r* = 0.87) with one with another. A similar phenomenon was observed in type 2 diabetes [80]. The correlation between salivary and serum oxidative biomarkers reinforces the utility of saliva as a valid diagnostic fluid. Since salivary ORAC can be a marker of systemic OS in IUGR, further studies are needed to confirm the utility of saliva in a clinical approach. We hypothesised that high salivary ORAC in IUGR may predict worse prognosis compared to IUGR patients without OS. If confirmed, IUGR patients with OS as a high-risk group may need to be monitored more intensively than IUGR patients without OS, e.g., more frequent ultrasound scans to confirm foetal wellbeing and planning for the time and route of delivery. This model of monitoring may reduce adverse perinatal outcomes.

## 5. Summary and Conclusion

Oxidative stress markers differ in two placental dysfunction pathologies: pregnancy-induced hypertension and IUGR. In this study, among patients with PIH, no sign of oxidative stress was observed. The major strength of this research is the analysis of the diet, inflammation markers, and oral health, which can strongly influence the oxidative status. To the best of our knowledge, this study is the first to assess OS in PIH using dietary data to interpret the results.

Oxidative stress was detected in patients with IUGR. Although no differences in the activity of antioxidant enzymes were found in plasma, the differences in the whole blood were observed.

The activity of blood ALDH in the IUGR group was 2-times lower than that in the control group, suggesting severe oxidative stress in the foretime. Higher ORAC in plasma of IUGR patients can be a compensatory mechanism and confirms the OS occurrence. Moreover, among patients with IUGR, a significantly higher activity of salivary ALDH was observed and this may be explained by the inflammation in the oral cavity within this group due to poor oral health status. Salivary MDA level is strongly influenced by the local environment of the oral cavity and should not be used as a marker of the systemic OS in patients with PIH and IUGR. On the contrary, salivary ORAC can be used to estimate ORAC in plasma in healthy pregnant women and in pregnancy pathologies like PIH or IUGR.

## Figures and Tables

**Table 1:** Oral health indicators.

P	Teeth with cavities
U	Missing teeth
W	Teeth with fillings
BI	Bleeding index: 0—healthy gums; 1—visually healthy, bleeding after probing; 2—change of colour, bleeding after probing; 3—change of colour, shape, bleeding after probing; 4—change of colour, shape, oedema, bleeding after probing; 5—change of colour, shape, spontaneous bleeding, oedema/ulceration
GI	Gingival index: 0—healthy gums; 1—inflammation, no bleeding, 2—mild inflammation, bleeding on probing, oedema; 3—severe inflammation, spontaneous bleeding, ulcerations
PD	Pocket depth
PI	Plaque index: Clean teeth Teeth with any plaque

**Table 2:** Selected clinical characteristics of the study population.

	PIH	IUGR	Controls
Number	27	30	47
Age range (years)	24-38	22-38	19-40
Gestational age^†^ (week)	36 (4)^a^	35.5 (4.5)^a^	39 (2)^a^
Gestational age range (week)	27-41	24-38	27-41
C-reactive protein^†^ (mg/l)	5.9 (13.8)^a^	3.6 (4.2)^a^	4.4 (3.6)^a^
White blood cells × 10^3^/*μ*l	10.5 (5.1)^b^	9.8 (4.3)^a^	9.3 (7.1)^ab^
Body mass index^†^ (kg/m^2^)	28.9 (7.4)^a^	24.6 (4.3)^a^	27.8 (5.4)^a^
Teeth with cavities^†^	3.0 (2.5)^a^	4.2 (2.6)^b^	2.6 (1.4)^a^
Missing teeth^†^	2.0 (1.4)^a^	3.3 (2.6)^b^	1.8 (1.0)^a^
Teeth with fillings^†^	6.7 (2.9)^a^	6.33 (1.7)^a^	4.2 (3.4)^a^
Bleeding index^†^	1.29 (0.49)^a^	1.71 (0.79)^a^	1.57 (0.76)^a^
Gingival index^†^	1.50 (0.58)^a^	1.88 (0.36)^b^	1.50 (0.53)^a^
Pocket depth^†^ (mm)	2.50 (0.76)^a^	2.7 (1.2)^a^	2.17 (0.58)^a^
Foetal weight^†^ (g)	2900 (740)^a^	1810 (1020)^b^	3360 (500)^c^
Apgar score^†^ (points)	9.0 (1.0)^a^	9 (1.5)^a^	10 (1.0)^a^
City inhabitant (% Warsaw)	41^a^	50^a^	34^a^

^abc^Homogenous group according to the Kruskal Wallis, ANOVA, or Chi-square tests. ^†^Results expressed as mean ± standard deviation or median ± interquartile range depending on data distribution.

**Table 3:** Energy and selected nutrient content (median and interquartile range) in the entire study population.

	PIH	IUGR	Controls
Energy (kcal)	1590 (740)^a^	1650 (590)^a^	1820 (570)^a^
From carbohydrates (%)	64 (13)^a^	65 (10)^a^	62 (12)^a^
From fats (%)	29.6 (8.6)^a^	26.9 (7.5) ^a^	30 (13) ^a^
From saturated fats (%)	13.4 (5.6)^a^	13.0 (6.1) ^a^	12.9 (7.4) ^a^
Flavonoids (mg)	630 (620)^a^	720 (510)^a^	660 (580)^a^
Flavonoids/kcal (*μ*g/kcal)	470 (300)^a^	430 (270)^a^	380 (280)^a^
Fibre/kcal (mg/kcal)	16.5 (7.3)^a^	13.5 (5.6)^a^	17.4 (6.8)^a^
Fibre (g)	26 (17)^a^	26 (8)^a^	27 (11)^a^
Vitamin E (mg)	7.2 (7.0)^a^	5.0 (2.7)^b^	7.9 (5.7)^a^
Vitamin C (mg)	130 (110)^a^	152 (160)^a^	119 (120)^a^
Folate (*μ*g)	280 (150)^a^	260 (120)^a^	310 (160)^a^
Heme-iron + supplementation (mg)	29 (47)^a^	28 (28)^a^	29 (31)^a^
Heme-iron^1^ (mg)	1.3 (1.5)^a^	1.2 (1.4)^a^	1.3 (0.8)^a^

^abc^Homogenous group according to the Kruskal Wallis test. ^1^Originating from food.

**Table 4:** Median and interquartile range of the entire study population.

	PIH	IUGR	Controls
Plasma GPx (U/ml)	29.9 (11)^a^	29.1 (8.6)^a^	28.8 (7.8)^a^
Plasma GST (U/ml)	29.8 (6.0)^a^	27.8 (9.5)^a^	28 (8.0)^a^
Plasma ORAC (mM)	17 (16)^ab^	20 (30)^a^	12 (13)^b^
Plasma MDA (*μ*M)	1.2 (1.3)^ab^	1.22 (0.84)^a^	1.53 (0.70)^b^
Salivary ALDH^1^ (U/g)	0.24 (0.40)^a^	0.34 (1.1)^b^	0.24 (0.36)^a^
Saliva ORAC (mM)	1.8 (2.6)^ab^	4.2 (4.1)^a^	1.5 (1.5)^b^
Blood ALDH^1^ (mU/g Hb)	7.1 (3.2)^b^	2.3 (5.9)^a^	4.7 (4.7)^b^
Saliva MDA (*μ*M)	0.32 (0.21)^a^	0.29 (0.34) ^a^	0.34 (0.18)^a^

^abc^Homogenous group according to the Kruskal Wallis test. ^1^Salivary ALDH and blood ALDH are different isoforms of the aldehyde dehydrogenase (i.e., isoforms 3 and 1, respectively) with different substrate specificities.

## Data Availability

The data used to support the findings of this study are available from the corresponding author upon request.
